# Wavelet Transform and Hierarchical Hybrid Matching for Enhancing End-to-End Pediatric Wrist Fracture Detection

**DOI:** 10.1007/s10278-025-01512-8

**Published:** 2025-05-30

**Authors:** Bin Yan, Yuliang Zhang, Qiuming He

**Affiliations:** 1 Department of Neonatal Surgery, Guangzhou Women and Children’s Medical Center, Guangzhou Medical University, National Children’s Medical Center for the South Central Region, No.9 Jinsui Road, Tianhe District, Guangzhou, 510623 Guangdong China; 2 Department of Obstetrics and Gynecology, The Third Affiliated Hospital of Guangzhou Medical University, No. 63 Duobao Road, Liwan District, Guangzhou, 510150 Guangdong China

**Keywords:** Fracture detection, DEtection TRansformer, End-to-end detector, Wavelet transform

## Abstract

With the increasing frequency of daily physical activities among children and adolescents, the incidence of wrist fractures has been rising annually. Without precise and prompt diagnosis, these fractures may remain undetected, potentially leading to complications. Recent advancements in computer-aided diagnosis (CAD) technologies have facilitated the development of sophisticated diagnostic tools, which significantly improve the accuracy of fracture detection. To enhance the capability of detecting pediatric wrist fractures, this study presents the WH-DETR model, specifically designed for pediatric wrist fracture detection. WH-DETR is configured as a DEtection TRansformer framework, an end-to-end object detection algorithm that obviates the need for non-maximum suppression post-processing. To further enhance its performance, this study first introduces a wavelet transform projection module to capture different frequency features from the feature maps extracted by the backbone. This module allows the network to effectively capture multi-scale and multi-frequency information, improving the detection of subtle and complex features in medical images. Secondly, this study designs a hierarchical hybrid matching framework that decouples the prediction tasks of different decoder layers during training, thereby improving the final predictive capabilities of the model. The framework improves prediction robustness while maintaining inference efficiency. Extensive experiments on the GRAZPEDWRI-DX dataset demonstrate that our WH-DETR model achieves state-of-the-art performance with only 43 M parameters, attaining an $\text {mAP}_{50}$ score of 68.8%, an $\text {mAP}_{50-90}$ score of 48.3%, and an F1 score of 64.1%. These results represent improvements of 1.78% in $\text {mAP}_{50}$, 1.69% in $\text {mAP}_{50-90}$, and 1.75% in F1 score, respectively, over the next best-performing model, highlighting its superior efficiency and robustness in pediatric wrist fracture detection.

## Introduction

Pediatric wrist fractures pose a considerable concern due to their frequency and significance in pediatric injuries. These injuries commonly involve fractures of the radius and ulna, especially during children’s active growth stages. Such fractures can impact their long-term development and functionality [1–3]. In recent years, the incidence of distal forearm fractures in children has been increasing due to the rising frequency of physical activities among children and adolescents. Studies indicate that distal forearm fractures account for 24% of fractures requiring hospitalization [6]. Given the close anatomical proximity and shared biomechanical stress between the distal forearm and the wrist, wrist fractures often co-occur with distal forearm fractures. As a result, precise and prompt diagnosis is vital to prevent long-term repercussions. However, this process is frequently fraught with challenges stemming from various factors, including a lack of medical professionals and limitations in technology and expertise [4, 5]. These difficulties are particularly acute in underdeveloped regions, where a paucity of radiologists exacerbates the challenges of timely diagnosis and medical care [4, 7].

In the realm of medical imaging, three primary modalities—X-ray, MRI, and CT scans—are employed for fracture diagnosis. Among these, X-ray is the most extensively utilized due to its cost-effectiveness [9], but its precision heavily relies on the radiologist’s proficiency. Research indicates that in pediatric X-ray fracture diagnosis, the most commonly overlooked fractures occur in the hand phalanges, with a miss rate reaching up to 26% [8]. Consequently, enhancing diagnostic accuracy and diminishing reliance on radiological expertise present significant challenges.

In recent years, the relentless advancement of deep learning technologies has led to significant progress in medical image processing applications [10–13], and computer-aided diagnosis (CAD) systems have made notable strides. An increasing number of researchers within the computer vision community are applying object detection algorithms to the task of identifying wrist fractures in children. These algorithms boost diagnostic accuracy and reduce labor costs by automating the analysis of medical images [14–19].

Nevertheless, these algorithms predominantly depend on models from the You Only Look Once (YOLO) series [20, 22–25], which incorporate numerous manually designed components, such as anchor generation, rule-based training target assignment, and non-maximum suppression (NMS) post-processing. These methods are not entirely end-to-end, and there is a significant risk of misdetection and missed detection due to inaccurate bounding box selection.

In contrast, models from the detection transformer (DETR) series [27, 28] eliminate the need for these manually designed components and have developed the first fully end-to-end object detector, achieving highly competitive performance. DETR models utilize a straightforward architecture that integrates convolutional neural networks (CNNs) with transformer [26] encoder-decoder networks. These models leverage the versatile and robust relational modeling capabilities of transformers without necessitating manual rule design.

**Fig. 1:**
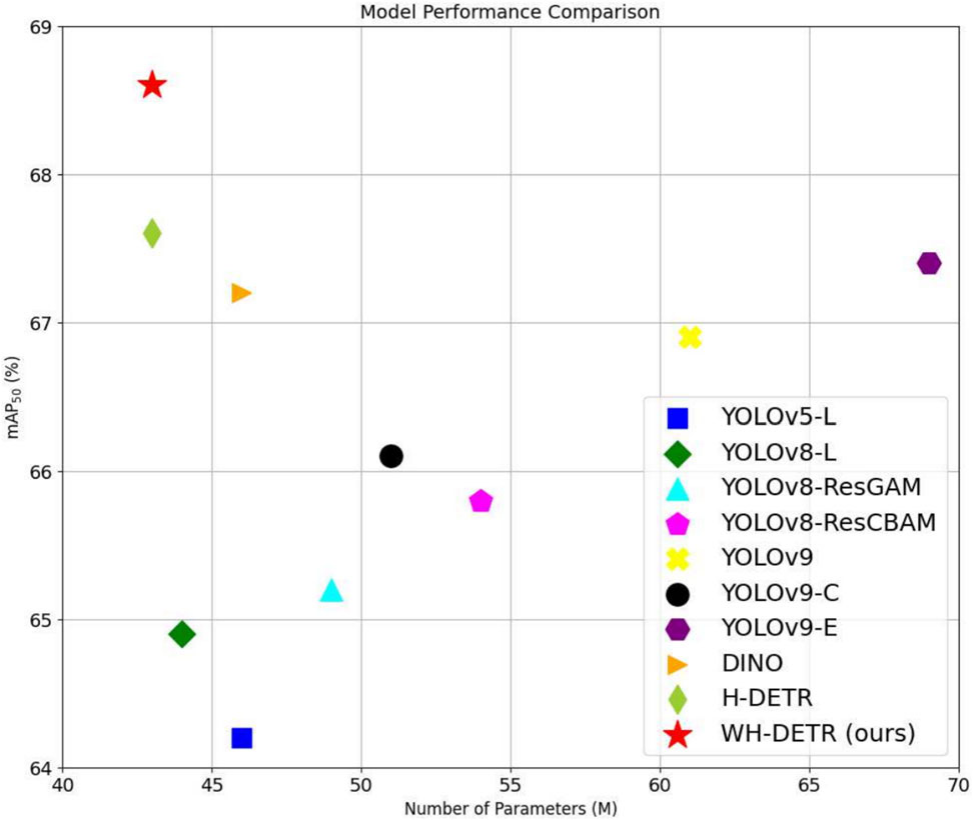
Comparison of various models. Each model is marked with a distinct marker. The red pentagram represents our proposed WH-DETR, which achieves the highest $\text {mAP}_{50}$ while maintaining the lowest parameter count

To capitalize on the advantages of DETR models and investigate their potential in other tasks, we adapt DETR models for the task of pediatric wrist fracture detection. Furthermore, we discovered that directly employing DETR models results in relatively inadequate performance. To rectify this, we propose two enhancements:

**1)** We incorporate a wavelet transform projection (WTP) module to capture varying frequency features from the feature maps extracted by the backbone. This assists the network in better capturing multi-scale and multi-frequency information from the images.

**2)** We devise a hierarchical hybrid matching (HHM) framework, which separates the prediction tasks of different decoder layers during training. This strategy enhances the model’s ultimate prediction capability without incurring any computational overhead during inference.

Building on these enhancements, we denominate the resulting model WH-DETR. As shown in Fig. 1, to substantiate the efficacy of our proposed model, we conducted comprehensive experiments on the GRAZPEDWRI-DX [32] dataset and compared our model with other sophisticated models, demonstrating the effectiveness of our proposed model. Additionally, we also present an in-depth discussion of the introduced WTP and HHM components through ablation experiments.

**Table 1:** Summary of literatures

Category	Method/model	Main contribution
Fracture detection	DCFPN [33]	mAP 82.1% for thigh fractures using dilated convolutions
	ParallelNet [34]	Two-stage R-CNN for thigh fractures
	FAMO [38]	Mitigated feature ambiguity for bone fracture detection
	DeepLoc [16]	Combined YOLOv7 with Swin Transformer for pediatric fractures
	YOLO Series [15]	Advanced YOLO models for pediatric wrist fracture detection
Transformer models	DETR [27]	End-to-end object detection with Transformer backbone
	DNDetr [47]	Improved training stability with denoising
	DINO [29]	Enhanced training efficacy with contrastive denoising
Wavelet transform	WT in DL [51]	Combined WT with DL for richer feature maps
Matching training	H-DETR [58]	Hybrid matching strategy for better positive samples

## Related Work

### Fracture Detection

The significance of fracture detection, combined with the evolution of deep learning (DL) technologies, has sparked a surge of interest among researchers in applying DL to this domain. Guan et al. [33] developed a DL model named the dilated convolutional feature pyramid network (DCFPN), which attained a mean average precision (mAP) of 82.1% in detecting thigh fractures from X-ray images. Wang et al. [34] introduced ParallelNet, a two-stage R-CNN [35] network, and compared its performance against other state-of-the-art DL models, such as Faster R-CNN [36] and Cascade R-CNN [37], on a dataset comprising 3842 thigh fracture X-ray images, showcasing its superiority. Wu et al. [38] proposed the Feature Ambiguity Mitigate Operator (FAMO) model, leveraging ResNeXt101 [39] and Feature Pyramid Network (FPN) [40], for bone fracture detection across 9040 radiographs of various body parts. Dibo et al. integrated YOLOv7 [23] with the Shifted Window Transformer (Swin) [41] to create DeepLoc, which achieved enhanced performance in pediatric wrist fracture detection. Additional studies [14, 15, 18, 42, 43] have also employed state-of-the-art YOLO series models to improve the accuracy of pediatric wrist fracture detection.

### Detection Transformer Models

In contrast to traditional object detection models, the detection transformer (DETR) [27] represents a novel transformer-based object detection model that minimizes dependence on manually designed components, thereby achieving true end-to-end detection. Many researchers have since proposed enhancements to DETR-like models [29–31, 44–49]. These improvements target various aspects such as the encoder, decoder, and training methodology, resulting in enhanced training efficiency, prediction accuracy, and inference speed.

### Wavelet Transform Algorithms

Wavelet transform (WT) [50], a signal processing technique widely utilized for time-frequency analysis, has recently been integrated with DL architectures across various tasks to bolster the performance of the underlying structures [51–55]. These studies emphasize the advantages of disentangling low-frequency and high-frequency components of the input for separate convolution operations, resulting in richer feature maps. Consequently, we incorporate WT into our DETR model to harness the rich information present in the frequency domain, thereby elevating the performance of our model.

### Hybrid Matching Training

The original DETR model utilizes a one-to-one matching strategy, pivotal for enabling end-to-end training. However, this approach results in only a minute fraction of the vast number of queries being matched and contributing to loss calculation, significantly hindering the model’s capacity to effectively train on positive samples. To mitigate this issue, DN-DETR [47] introduces noise into the ground truth bounding boxes, training the decoder to eliminate these disturbances. This denoising process aids network training and accelerates convergence. Similarly, DINO [29] improves network training by adopting a denoising mechanism akin to DN-DETR, employing contrastive denoising to enhance training efficacy. Although these methods still adhere to the one-to-one matching principle, they integrate denoising with standard training, thereby improving training stability. H-DETR [58] addresses the challenge of inadequate positive sample training by combining the original one-to-one matching branch with an auxiliary one-to-many matching branch during training. This hybrid training strategy enhances model performance by ensuring more effective utilization of positive samples.

To better present these literatures, we summarize them in Table 1, where the main contributions of each literature are concisely listed.

**Fig. 2:**
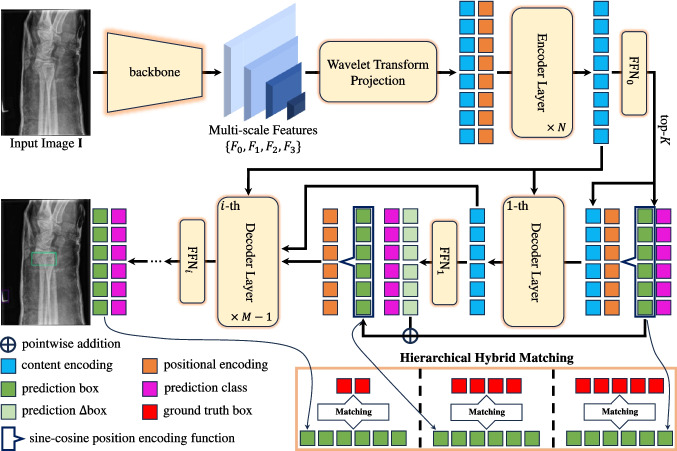
The framework of WH-DETR. Drawing inspiration from Deformable DETR [28], our approach utilizes a backbone to extract multi-scale features from the input image. Additionally, we apply the wavelet transform projection (WTP) to all scale features to encapsulate multi-frequency information. Throughout the model training phase, our hierarchical hybrid matching (HHM) framework facilitates matching between prediction anchors from different decoder layers and a varying number of ground truth anchors for loss computation. It is important to note that this diagram serves as a conceptual illustration; in practice, both prediction boxes and prediction classes are involved in the matching process

## Methodology

### Model Framework

DETR-like models typically comprise four essential components: a backbone, a multi-layer transformer [26] encoder, a multi-layer transformer decoder, and a feed-forward network (FFN). As illustrated in Fig. 2, our model architecture incorporates an additional projection component, corresponding to our proposed wavelet transform projection (WTP) module. The following sections offer detailed explanations of each component.

**Backbone:** The backbone of DETR, denoted as $\mathcal {F}_b$, extracts preliminary features from the input image. In this study, we employ ResNet50 [56] as the backbone, which has proven to be an effective feature extractor in numerous studies. For an input image $\textbf{I} \in \mathbb {R}^{3 \times \textsf{H} \times \textsf{W}}$, the backbone outputs four lower-resolution feature maps $\{F_0, F_1, F_2, F_3\}$, with spatial dimensions $H_i$ and $W_i$ scaled down by factors of $\frac{1}{4^2}$, $\frac{1}{8^2}$, $\frac{1}{16^2}$, and $\frac{1}{32^2}$ relative to the original image, respectively, and channel dimensions $C_i$ of 256, 512, 1024, and 2048, respectively. This can be concisely expressed as follows:

1 $$\begin{aligned} \{F_0, F_1, F_2, F_3\} = \mathcal {F}_b(\textbf{I}) \end{aligned}$$

**Fig. 3:**
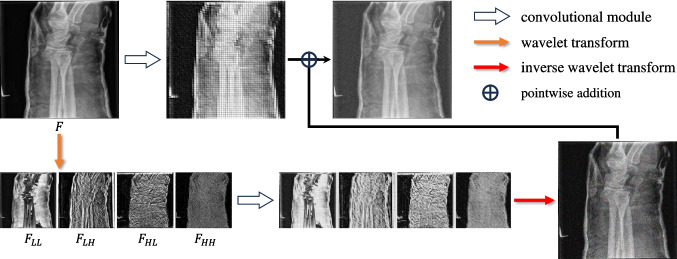
Details of a single-level WTP module. The input feature and subband features are depicted as examples, not reflecting the actual features within the model

**Projection Layer:** The projection layer, denoted as $\mathcal {F}_p$, transforms the feature maps from the backbone into the input format required by the transformer encoder. As shown in Eq. 2, this layer first processes the multi-scale feature maps to unify their channel dimensions, set to 256 in our experiments. These processed feature maps are then concatenated along the channel dimension to form the content encoding $\textbf{C}_e \in \mathbb {R}^{256 \times \sum H_i W_i}$.

2 $$\begin{aligned} \textbf{C}_e = \mathcal {F}_p(\{F_0, F_1, F_2, F_3\}) \end{aligned}$$

**Feed-Forward Network:** For clarity, we introduce the concept of a feed-forward network before discussing the transformer encoder and decoder in detail. Our model comprises a total of $M+1$ FFN layers: $\text {FFN}_e$ corresponds to the final layer of the encoder, while $\text {FFN}_1$ to $\text {FFN}_M$ correspond to the *M* decoder layers. Each FFN layer consists of a three-layer MLP network with ReLU activation, followed by an additional linear layer. The MLP network predicts bounding box offsets, while the linear layer predicts the corresponding class labels.

**Transformer Encoder:** The transformer encoder, denoted as $\mathcal {F}_e$, consists of a stack of *N* transformer blocks, with $N=6$ in this study. Its role is to enhance the understanding of the content encoding. For the input $\textbf{C}_e$, positional encoding $P_e$ [27] is generated and added, which is then fed into the transformer encoder to produce the enhanced content encoding $\dot{\textbf{C}_e}$. The output $\dot{\textbf{C}_e}$ is subsequently passed to a $\text {FFN}_0$, which predicts the offsets of bounding boxes ${\delta b}_e$ and their corresponding classes $c_e$. In this study, we adopt the method from DINO [29] to generate the initial decoder bounding boxes: $b_e = b'_e + {\delta b}_e$, where $b'_e$ are initialized with a uniform distribution over the input image. Given that the token count of $\dot{\textbf{C}_e}$ is $\sum H_i W_i$, we select the top-*K* predictions based on confidence scores from $c_e$:

3 $$\begin{aligned} \textbf{C}_d^0, b_d^0, c_d^0= &   \text {top-}K(\dot{\textbf{C}_e}, b_e, c_e)\end{aligned}$$

4 $$\begin{aligned} \dot{\textbf{C}_e}= &   \text {FFN}_0(\textbf{C}_e) \end{aligned}$$

where $K=300$ and $\textbf{C}_d^0, \textbf{b}_d^0, \textbf{c}_d^0$ are the selected results, which serve as the input to the transformer decoder.

**Transformer Decoder:** The transformer decoder, denoted as $\mathcal {F}_d$, comprises *M* transformer blocks, with $M=6$ in this study. Following standard transformer input protocols, we use a sine-cosine position encoding function [26] to generate positional encodings for each block input:

5 $$\begin{aligned} \textbf{P}_d^i= &   \text {sinusoidal}(\text {sigmoid}(b_d^i)), \; i \in \{0, 1, 2, \ldots , M -1\}\nonumber \\ \end{aligned}$$

6 $$\begin{aligned} b_d^i= &   b_d^{i-1} + {\delta b}_d^i, \; i \in \{1, 2, \ldots , M\}\end{aligned}$$

7 $$\begin{aligned} {\delta b}_d^i, c_d^i= &   \text {FFN}_i(\textbf{C}_d^{i}), \; i \in \{1, 2, \ldots , M\}\end{aligned}$$

8 $$\begin{aligned} \textbf{C}_d^{i}= &   \mathcal {F}_d^i(\textbf{C}_d^{i-1}, \textbf{P}_d^{i-1}, \dot{\textbf{C}_e}), \; i \in \{1, 2, \ldots , M\} \end{aligned}$$

where $\mathcal {F}_d^i$ denotes the *i*-th decoder block (for $i > 0$) and $\text {FFN}_i$ represents the corresponding feed-forward network. To optimize the parameters of adjacent transformer decoder layers more effectively, we also incorporate the look forward twice scheme [29].

To expedite our model, we employ the deformable attention mechanism proposed in Deformable DETR [28]. This mechanism concentrates attention on a small set of critical sampling points around a reference, rather than computing attention over all inputs, significantly reducing the overall computational cost of the model.

### Wavelet Transform Projection

The single-level WTP module’s details are illustrated in Fig. 3. Given an input feature *F*, we adopt the methodology outlined in [55, 57], applying a 2D Haar Wavelet Transform (WT). Specifically, four filters are employed:

9 $$\begin{aligned} \begin{aligned} f_{LL}&= \frac{1}{2} \begin{bmatrix} \quad 1 &  \quad 1 \\ \quad 1 &  \quad 1 \end{bmatrix},&f_{LH}&= \frac{1}{2} \begin{bmatrix} \quad 1 &  -1 \\ \quad 1 &  -1 \end{bmatrix} \\ f_{HL}&= \frac{1}{2} \begin{bmatrix} \quad 1 &  \quad 1 \\ -1 &  -1 \end{bmatrix},&f_{HH}&= \frac{1}{2} \begin{bmatrix} \quad 1 &  -1 \\ -1 &  \quad 1 \end{bmatrix} \end{aligned} \end{aligned}$$

Here, $f_{LL}$ is a low-pass filter for extracting low-frequency features. $f_{LH}$ functions as a low-pass filter horizontally and a high-pass filter vertically. Similarly, $f_{HL}$ operates as a low-pass filter vertically and a high-pass filter horizontally, while $f_{HH}$ acts as a high-pass filter in both directions. Convoluting these filters with *F* across channels yields four corresponding subband features. Due to the stride of 2 in convolution, the spatial resolution of the resulting feature maps is halved. To revert the decomposed features to their original resolution, an inverse wavelet transform can be employed, implemented via transposed convolution. Thus, the two processes can be represented as follows:

10 $$\begin{aligned} [F_{LL}, F_{LH}, F_{HL}, F_{HH}] = g([f_{LL}, f_{LH}, f_{HL}, f_{HH}], F) \end{aligned}$$

11 $$\begin{aligned} \begin{aligned} F = g_t(&[f_{LL}, f_{LH}, f_{HL}, f_{HH}],\\&[F_{LL}, F_{LH}, F_{HL}, F_{HH}]) \end{aligned} \end{aligned}$$

where $g(\cdot )$ and $g_t(\cdot )$ denote convolution and transposed convolution operators, respectively.

To bolster feature representation, the original feature map *F* and the subband features $F_{LL}$, $F_{LH}$, $F_{HL}$, and $F_{HH}$ derived from the wavelet transform are each fed into a corresponding learnable convolution module. These modules further extract and integrate new feature information. The final feature map $F'$ is obtained via the following formula:

12 $$\begin{aligned} F' = \mathcal {F}_{\text {conv}}(F) + \text {IWT}(\mathcal {F}_{\text {conv}}'(\text {WT}(F))) \end{aligned}$$

Here, $\text {WT}(\cdot )$ and $\text {IWT}(\cdot )$ correspond to Eqs. 10 and 11, respectively, while $\mathcal {F}_{\text {conv}}$ and $\mathcal {F}_{\text {conv}}'$ represent two learnable convolution modules with a kernel size of 5.

For multi-level WTP, the process involves sequentially applying WT and IWT to the low-frequency subband feature $F_{\text {LL}}$ produced by the WT decomposition of the previous level. After these transformations, the updated $F_{\text {LL}}'$ replaces the original $F_{\text {LL}}$ for subsequent operations.

It is crucial to note that the filter parameters used in WT and IWT are independent. Although both sets of parameters are initialized using Eq. 9, the WT parameters remain fixed, whereas the IWT parameters are adjusted by the model during training. This distinction is necessary because the features undergo non-linear transformation through $\mathcal {F}'_{\text {conv}}$ after WT. Therefore, it is imperative for the model to adjust the IWT parameters to prevent feature loss after applying IWT.

### Hierarchical Hybrid Matching

Similar to [58], we incorporate one-to-many matching to enhance the model’s effective training on positive samples. Specifically, we compute auxiliary losses for the outputs of the final encoder layer and the *M* decoder layers. However, for each layer, we employ a varying number of one-to-many matches when calculating these auxiliary losses. This variation is due to the fact that shallower Transformer decoder layers may not fully capture cross-correlations between output elements, leading to multiple predictions for the same object. As the layers deepen, the self-attention mechanism increasingly aids the model in suppressing duplicate predictions [27]. Consequently, using too few matches in the first layer might cause the model to misinterpret many nearly correct predictions. Conversely, employing a large number of one-to-many matches in deeper decoder layers could impede the model’s ability to learn to suppress duplicates, thereby affecting overall training performance.

Based on this analysis, our proposed HHM is designed to gradually decrease the number of one-to-many matches as the decoder layers deepen. Specifically, we define $\textbf{p}_i = \{b^i_d, c^i_d\}$ as the prediction results of the *i*-th decoder layer (with $i=0$ denoting the encoder’s predictions), and $\textbf{g}$ as the ground truth annotations. Our loss function can thus be expressed as follows:

13 $$\begin{aligned} \mathcal {L}= &   \sum _{i=0}^{M-1} \frac{1}{w_i} \mathcal {L}_\text {Hungarian}(\textbf{p}_i, copy(\textbf{g}, w_i)) \nonumber \\  &   + \mathcal {L}_\text {Hungarian}(\textbf{p}_M, \textbf{g})) \end{aligned}$$

Here, $\mathcal {L}_\text {Hungarian}$ represents the Hungarian loss [27], comprising a classification loss, an $L_1$ regression loss, and a GIoU loss. Additionally, $copy(\textbf{g}, w_i)$ indicates duplicating the ground truth $\textbf{g}$ $w_i$ times. In our experiments, with $M=6$, the values of $w_0$ to $w_5$ are set to [12, 10, 8, 6, 4, 2].

This straightforward design allows us to decouple the prediction tasks across different decoder layers. Shallower decoders are tasked with locating approximate object positions, resulting in a larger number of candidate bounding boxes. In contrast, deeper decoders refine these positions more accurately by suppressing duplicate predictions, thus providing more precise predictions from a broader set of candidates.

**Fig. 4:**
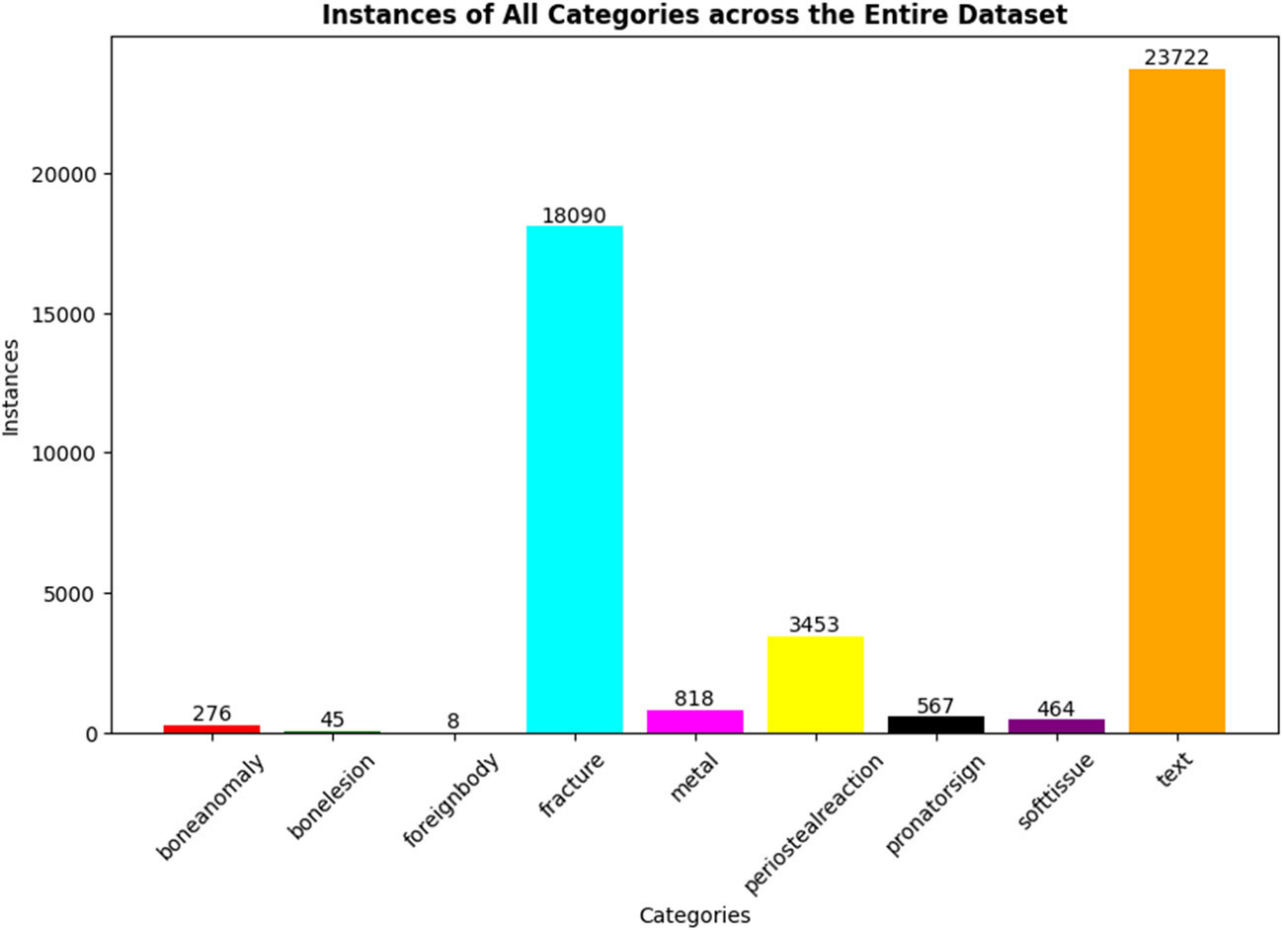
A bar chart illustrating the statistical count of instances across all categories within the entire dataset

## Experiment

### Dataset

For this study, we utilized the publicly accessible GRAZPEDWRI-DX dataset [32], kindly provided by the Medical University of Graz. This dataset comprises 20,327 X-ray images of pediatric wrist trauma. Figure 4 displays the distribution of instances among all categories, revealing a notable class imbalance. Specifically, the *text* and *fracture* categories account for 50.00% and 38.13% of the total instances, respectively, while the remaining seven categories collectively constitute only 11.73%.

It is important to note that all images are sourced from 6091 unique pediatric patients. During model training, we assume that the similarity between multiple images from the same patient can be neglected. To verify this assumption, we utilize the backbone of the trained model to generate feature maps for each image and subsequently pass them through a max-pooling layer to obtain features, which are generally considered to capture the most salient features of the model. For these features, we compute the cosine similarity matrix and then count how many features have their most similar counterpart belonging to the same patient. The results indicate that only 1.87% of the total features (323/17,278, using only the training set) have their most similar counterpart belonging to the same patient. In fact, since the subsequent model employs these features, we can consider the image correlation in this dataset to be negligible in our work.

### Experimental Setup

In our experimental setup, we randomly divided the entire dataset into 85% for training (17,278 images) and 15% for testing (3047 images). During the training phase, we employed basic data augmentation techniques such as random horizontal flipping, random resizing, and random cropping. No data augmentation was applied during testing. Given that the X-ray images in the GRAZPEDWRI-DX dataset are single-channel with pixel values ranging from 0 to 65,535, we normalized these pixel values and replicated the channel three times to create RGB format inputs.

All experiments were carried out using two NVIDIA GeForce RTX 4090 GPUs. Due to resource constraints, the batch size utilized during training was set to 2. We employed the AdamW [59] optimizer, initializing the learning rate at $10^{-5}$ for the backbone and $10^{-4}$ for other parameters. These learning rates were subsequently reduced to one-tenth of their initial values at the 10th and 30th epochs. The weight decay was set to $10^{-4}$, and the dropout rate was maintained at 0. The number of attention heads in the transformer was configured to 8, with an attention feature dimension of 256. The loss function weights used in our experiments were consistent with those in [27]. Our experimental procedure encompassed a total of 50 training epochs, which were completed in approximately 20 h.

Due to the DETR class model’s direct output of a number of boxes equal to the number of queries, our experimental setup specifies that the model will produce 300 candidate boxes, each accompanied by a probability for the predicted category. We retain only those results with a probability greater than 0.3, since the probability associated with predicted categories corresponding to unmatched queries is generally low.

**Fig. 5:**
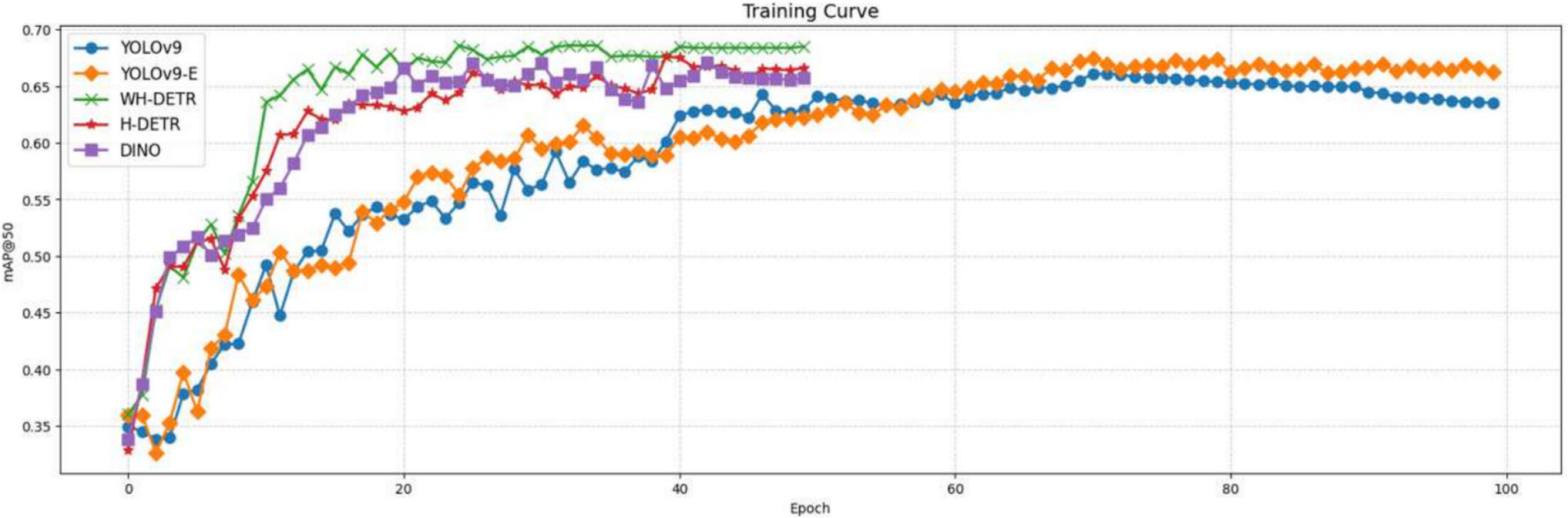
Comparison of training curves between YOLO series and DETR series

### Evaluation Metrics

The main evaluation metrics utilized in this study are outlined below:

- **Number of parameters (Params):** The number of parameters within a model reflects its architectural complexity, influenced by the quantity of layers and neurons per layer. A larger number of parameters typically signify a more extensive model, which may yield better performance but also necessitates more data and computational resources. Consequently, achieving a balance between model complexity and computational cost is crucial in practical applications.
- **Floating point operations (FLOPs):** FLOPs serve as a measure of the computational complexity of neural network models and are a standard metric for assessing computer or computing system performance. FLOPs denote the number of floating-point operations executed per second, acting as a key indicator of a model’s computational efficiency and speed. In resource-limited settings, models with lower FLOPs may be preferable, whereas those with higher FLOPs might require more robust hardware support.
- **Mean average precision at 50% IOU (**${\textbf {mAP}}_{{\textbf {50}}}$**):** mAP is a widely adopted metric for evaluating object detection model performance. In object detection tasks, models must identify objects within images and ascertain their locations. mAP integrates precision (the proportion of detected objects that are genuine) and recall (the proportion of actual objects detected). Specifically, mAP50 refers to the mean average precision calculated at a 50% Intersection over Union (IoU) threshold across all categories. IoU gauges the overlap between predicted and ground truth bounding boxes.
- ${\textbf {mAP}}_{{\textbf {50-90}}}$**:** This metric offers a more comprehensive assessment by considering the mean average precision across IoU thresholds ranging from 50 to 95%. mAP$_{50-90}$ is computed by determining the average precision at ten distinct IoU thresholds, incrementing from 0.50 to 0.95 in steps of 0.05, and subsequently averaging these AP values. This metric provides a stricter and more meaningful evaluation of model performance.
- **Additional classification metrics:** To facilitate a more robust and interpretable analysis of the experimental results, we employed the **F1** score and **AUC** as supplementary evaluation metrics. The F1 score, which harmonizes precision and recall, provides a balanced assessment of a model’s classification efficacy, particularly in scenarios involving class imbalance. This metric mitigates the potential bias associated with an exclusive focus on either precision or recall. The area under the curve (AUC) quantifies the area under the receiver operating characteristic (ROC) curve, which delineates the trade-off between the true positive rate and the false positive rate across varying classification thresholds. An AUC value approaching 1 signifies superior classification performance. Since our task contains multiple categories, we first calculate the metrics for each category and then take the average of these metrics as the final result.

**Table 2:** Comparison results between WH-DETR and other advanced object detector

Model	Params (M)	FLOPs (G)	$\text {mAP}_{50}$ (%)	$\text {mAP}_{50-90}$ (%)	F1 (%)	AUC (%)
YOLOv5-L	46	**109**.**1**	64.2	43.5	58.8	70.4
YOLOv8-L	44	164.9	64.9	43.9	59.2	72.0
YOLOv8-ResGAM	49	183.5	65.2	43.8	60.9	74.8
YOLOv8-ResCBAM	54	164.9	65.8	45.6	61.6	76.2
YOLOv9-C	51	239.0	66.1	45.9	61.8	78.3
YOLOv9	61	266.2	66.8±1.2	46.6±0.6	61.2±2.4	78.3±3.1
YOLOv9-E	69	244.9	67.5±1.3	47.1±0.8	63.0±3.0	79.2±3.1
DINO	46	238.1	67.1±1.3	47.3±0.6	62.5±2.6	**81.8±2.4**
H-DETR	**43**	223.5	67.6±1.4	47.5±0.7	61.0±3.0	81.3±2.5
WH-DETR (ours)	**43**	223.5	**68.8±1.2**	**48.3±0.7**	**64.1±2.8**	81.6±3.0

### Comparative Experiments

Our primary focus was on comparing models from the YOLO series and the DETR series. Numerous models in the YOLO series are regarded as state of the art (SOTA), and the DETR series we examined are also at the cutting edge of technology. In our experiments, the YOLO series models were used with their default settings, including pre-trained weights, specific data processing techniques, and parameter configurations. To ensure a fair comparison, all DETR-like models used ResNet50 as their backbone and pre-trained weights from the MS COCO (Microsoft Common Objects in Context) 2017 Detection task [21]. Given that the pre-trained categories differed from those in our task, we randomly initialized layers with mismatched parameter dimensions. Notably, H-DETR builds on our base model by integrating the one-to-many matching branch they introduced.

Figure 5 shows the training curves of several YOLO models and DETR models. Given the differences in their loss functions, we select $\text {mAP}_{{\textbf {50}}}$ as the *y*-axis for display. From the results of this display, we can see that DETR models can usually converge within 50 training epochs. Therefore, in the entire experiment, YOLO series models are trained for 100 epochs , while DETR models are trained for 50 epochs.

To reduce experimental costs, we selected only the four most comparable models for statistical analysis. Specifically, to ensure the statistical significance of the results, each model was trained 5 times. The first training session was conducted fully, while the subsequent sessions were initialized using the parameters from the first training, with the addition of Gaussian noise with zero mean and a standard deviation of 5% of the parameter values. This approach significantly reduced training time, as models typically converged within 15 epochs, while maintaining the validity of the experiments through the introduced randomness.

**Table 3:** Results of the one-way ANOVA test

$\text {mAP}_{50}$	$\text {mAP}_{50-90}$	F1	AUC
0.0006	<0.0001	0.0101	0.0027

The comparison results between our proposed WH-DETR and other advanced object detection methods are presented in Table 2. The best results are highlighted in bold, and the “±” values indicate the standard deviation.

To statistically examine the significant differences in these results, we first conducted a one-way ANOVA test. As shown in Table 3, each cell in the table presents the *p*-value obtained from the ANOVA test for the corresponding metric. For each metric with a *p*-value threshold of 0.05, we observed significant differences among the models.

**Table 4:** Post hoc Tukey HSD test results

WH-DETR vs. models	$\text {mAP}_{{50}}$	$\text {mAP}_{{50-90}}$	F1	AUC
YOLOv9	$\boldsymbol{<0.001}$	$\boldsymbol{<0.001}$	**0.0293**	0.1914
YOLOv9-E	**0.0705**	$\boldsymbol{<0.001}$	0.7899	0.1578
DINO	**0.0044**	$\boldsymbol{<0.001}$	**0.4731**	0.9997
H-DETR	0.1232	**0.0045**	**0.0163**	0.9985

**Table 5:** Effect size analysis using Cohen’s *d*

WH-DETR vs. Models	$\text {mAP}_{{50}}$	$\text {mAP}_{{50-90}}$	F1	AUC
YOLOv9	1.46$^{\S }$	2.54$^{\S }$	0.89$^{\S }$	0.90$^{\S }$
YOLOv9-E	0.86$^{\S }$	1.56$^{\S }$	0.31$^{\dagger }$	0.65$^{\ddagger }$
DINO	1.17$^{\S }$	1.50$^{\S }$	0.48$^{\dagger }$	0.07$^{\dagger }$
H-DETR	0.75$^{\ddagger }$	1.11$^{\S }$	0.88$^{\S }$	0.11$^{\dagger }$

The symbols $^{\dagger }$, $^{\ddagger }$ , and $^{\S }$ denote small, medium, and large effects, respectively

**Table 6:** Detection results for each category

Category	Instances	Precision (%)	Recall (%)	$\text {mAP}_{{50}}$ (%)	$\text {mAP}_{{50-90}}$ (%)	F1 (%)	AUC (%)
All	7187	71.0	61.8	68.8	48.3	64.1	81.6
Boneanomaly	52	47.8	28.8	35.9	23.0	35.9	65.9
Bonelession	7	57.1	47.8	55.5	33.7	52.0	80.3
Foreignbody	4	50.0	37.5	43.5	35.8	42.9	81.4
Fracture	2744	91.0	94.1	94.8	64.2	92.5	91.4
Metal	118	99.1	81.5	94.1	85.9	89.4	96.6
Periostealreaction	530	76.5	65.0	74.0	42.1	70.3	75.5
Pronatorsign	83	49.7	79.2	71.4	41.4	61.1	81.5
Softtissue	73	70.4	23.4	51.2	33.6	35.1	70.9
Text	3576	97.2	98.8	98.9	75.3	98.0	96.0

We further performed the Tukey HSD analysis. The adjusted *p*-values (p-adj) between WH-DETR and the other four models are listed in Table 4. Each cell represents the p-adj for the corresponding model and metric. With a p-adj threshold of 0.05, which is a more common threshold in statistical analysis, values in bold indicate significant differences between the models. The results show that WH-DETR exhibits significant differences from the other models across all metrics except **AUC**.

Finally, we conducted an effect size analysis using Cohen’s *d*, calculated as follows:

14 $$\begin{aligned} {d = \frac{\bar{X}_1 - \bar{X}_2}{s_p}} \end{aligned}$$

where the pooled standard deviation $s_p$ is given by:

15 $$\begin{aligned} s_p = \sqrt{\frac{(n_1 - 1)s_1^2 + (n_2 - 1)s_2^2}{n_1 + n_2 - 2}} \end{aligned}$$

The sample variance was computed using an unbiased estimator (denominator $n-1$). The results, presented in Table 5, were evaluated against thresholds of 0.3, 0.5, and 0.8, corresponding to small, medium, and large effects, respectively. The analysis indicates that most comparative experiments exhibit large effects.

The experiments reveal that WH-DETR achieves the best performance in terms of **Params**, **mAP**$_{{\textbf {50}}}$, **mAP**$_{\boldsymbol{50-90}}$ and **F1**. Although WH-DETR does not achieve the best performance in **AUC**, statistical analysis indicates that there is no significant difference between WH-DETR and the other compared models in terms of **AUC**. For the comparison of **FLOPs**, we resized the inputs of all models to $640 \times 640$. The findings indicate that our model has higher **FLOPs**, which can be attributed to the computationally demanding self-attention mechanism in transformers [26].

We discovered that DETR-based models surpass YOLO-based models in terms of **mAP**$_{{\textbf {50-90}}}$. This is due to the YOLO series modifying the boundaries of predicted boxes during post-processing based on manually set rules. If these rules are not accurate, it may result in offsets in the bounding boxes, whereas DETR-like methods directly predict object bounding boxes, thereby avoiding this issue.

### Detailed Experimental Results

#### Quantitative Experimental Results

**Fig. 6:**
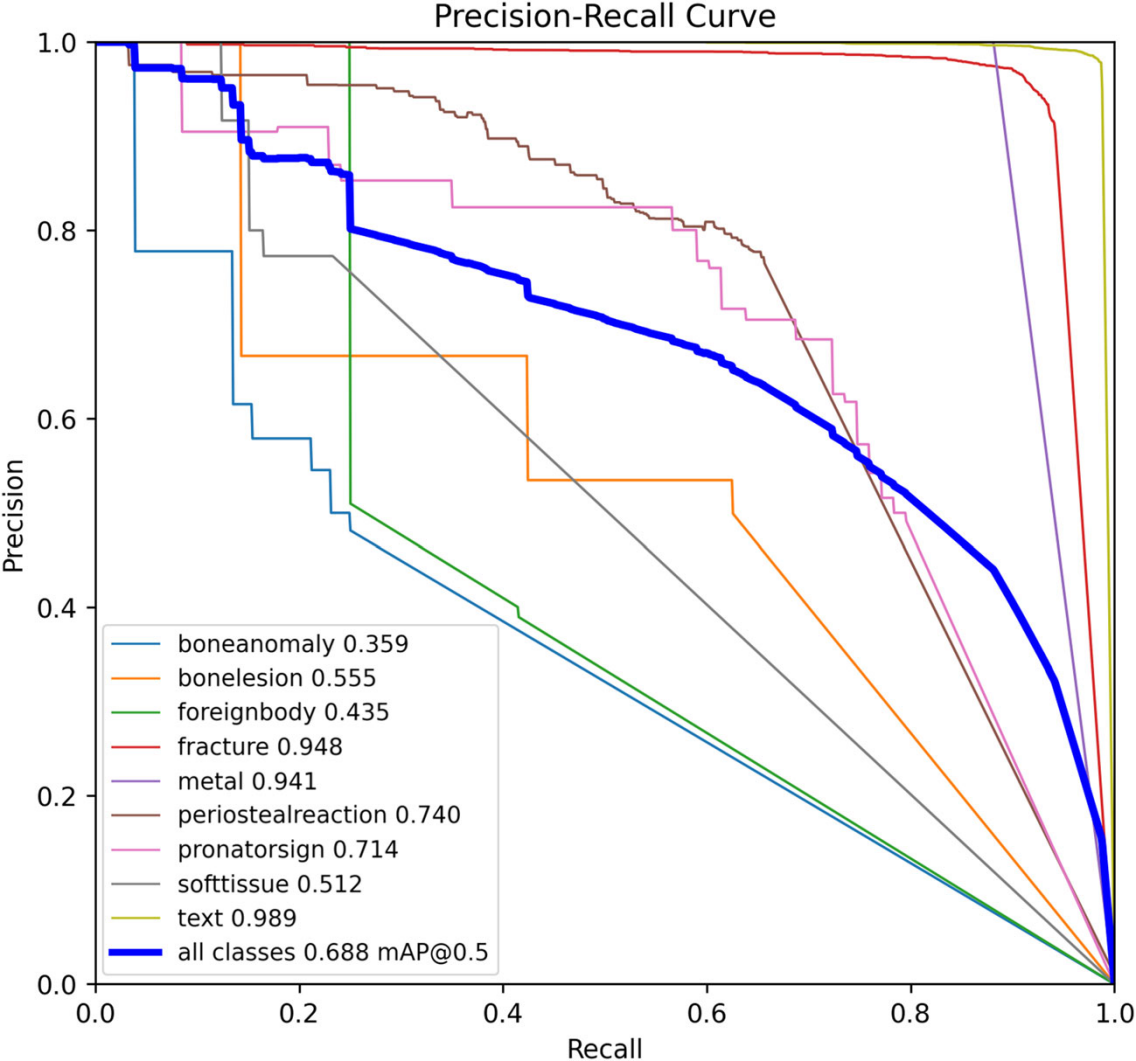
Precision-recall curves of each class using the WH-DETR model

To provide a more comprehensive understanding of the model’s performance, we present our quantitative experimental results. Table 6 displays the detection results for each category by WH-DETR, and Fig. 6 illustrates the precision-recall curves. Both precision and recall measurements employ an IOU threshold of 0.5. Our experimental results reveal that our model demonstrates superior performance in the *text*, *fracture*, and *metal* categories. This can be attributed to the larger number of instances in the first two categories, as well as the distinctive characteristics inherent in the third category. This observation implies that increasing the dataset size for the remaining categories may further enhance our model’s overall performance.

**Fig. 7:**
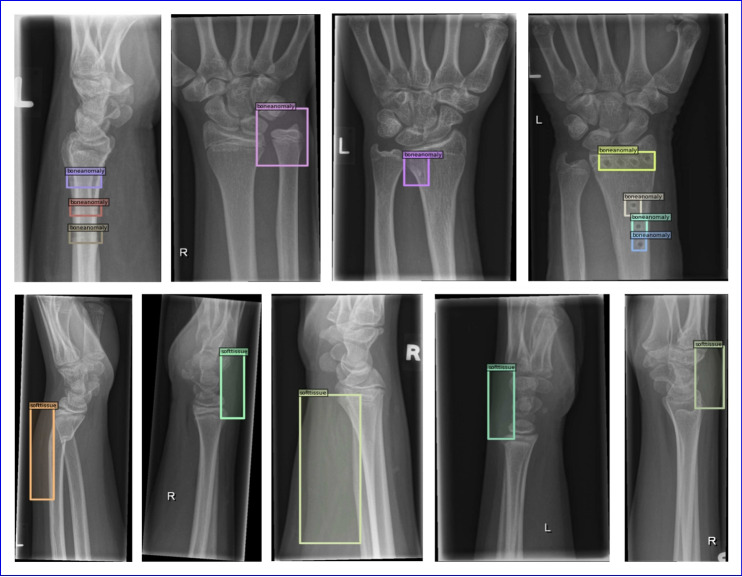
Examples of annotation for *boneanomaly* and *softtissue*

Concurrently, our model performs worst in the *boneanomaly* and *softtissue* categories. To elucidate this phenomenon, we provide the annotated examples for these two disease categories in Fig. 7. The category of bone anomaly represents a heterogeneous disease group, characterized by a diverse range of specific disease types and etiological factors. The complexity of this disease category means that its features are multifaceted. As a result, accurate identification solely through X-ray imaging is challenging. Therefore, increasing the accuracy of diagnosis can be achieved through methods such as oral inquiries. Similarly, the characteristic of soft tissue, being less dense than bone, leads to stronger X-ray penetrability and insufficient contrast. The feature of soft tissue in X-ray images is often less distinct compared to that of bone. This requires the integration of other imaging modalities, such as ultrasound and magnetic resonance imaging (MRI), for a more accurate and definitive diagnosis.

#### Qualitative Experimental Results

**Fig. 8:**
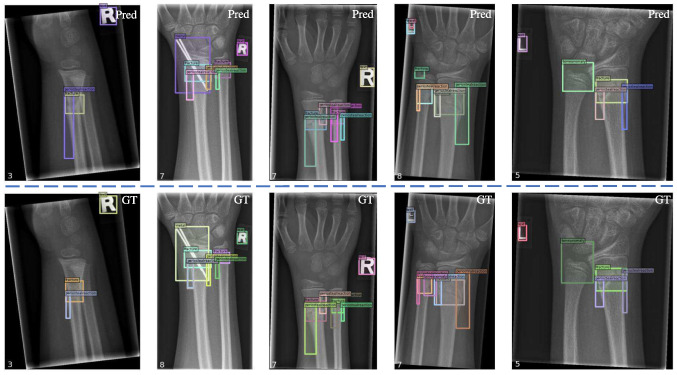
Qualitative experimental results. The top row displays the predictions made by our model, while the bottom row represents the ground truth. The number of bounding boxes is indicated in the bottom-left corner of each image

In order to more intuitively represent the outcomes of our model, we showcase our quantitative experimental results in Fig. 8. All presented results were obtained from the test set. In the illustrated examples, the predicted category and predicted candidate box for each instance are very close to the ground truth (GT). Moreover, in the second prediction, there are two overlapping *periostealreaction* in GT (gray and light blue), which our model predicts as a single entity, resulting in one fewer prediction than GT. In the fourth prediction result, we correctly predicted two *texts*, but one annotation was missing from the data. These examples illustrate that our model can predict more accurately than manually annotated results in certain cases, highlighting its strong generalization capability.

**Table 7:** Ablation results for each component

WTP	HHM	Params (M)	FLOPs (G)	$\text {mAP}_{{50}}$ (%)	$\text {mAP}_{{50-90}}$ (%)	F1 (%)	AUC (%)
✗	✗	46	232.3	66.7±1.0	47.2±0.7	61.2±1.6	77.6±3.1
✓	✗	43	223.5	67.4±1.4	47.6±0.6	62.5±2.7	79.2±2.8
✗	✓	46	232.3	68.1±0.9	47.8±0.5	62.9±3.0	79.7±2.4
✓	✓	43	223.5	**68.8±1.2**	**48.2±0.7**	**64.1±2.8**	**81.6±3.0**

Bold entries highlight the best-performing metrics

### Ablation Experiments

To reduce the cost associated with ablation experiments, we employ an approach analogous to that used in the comparative experiments. Specifically, during the initial run, the model undergoes full training. In subsequent runs, the model parameters are initialized by leveraging the weights obtained from the first training, with added noise. For the modules slated for removal, we bypass their parameter copying procedure entirely. Regarding the additional modules, we utilize the default initialization weights. All ablation experiments are repeated 5 times, and the model is evaluated based on the mean and standard deviation of the experimental results.

#### Impact of Each Component

To assess the contribution of each component to our model, we conducted ablation experiments for each component. Table 7 displays the effectiveness of the components we incorporated. When WTP is not utilized, we replace it with a convolution block with a kernel size of 3 to maintain comparable **Params** and **FLOPs**. The results indicate that incorporating WTP enhances the ${\textbf {mAP}}_{\boldsymbol{50}}$, ${\textbf {mAP}}_{\boldsymbol{50-90}}$, **F1**, and **AUC** by 0.7%, 0.4%, 1.3%, and 1.6%, respectively. This demonstrates that WTP effectively captures multi-frequency features of the input, thereby improving model performance. Furthermore, although HHM does not affect **Params** and **FLOPs**, it significantly benefits the model’s training process, leading to a 1.4%, 0.6%, 1.7%, and 2.1% in ${\textbf {mAP}}_{\boldsymbol{50}}$, ${\textbf {mAP}}_{\boldsymbol{50-90}}$, **F1**, and **AUC**, respectively.

**Table 8:** Performance of WTP under different experimental settings

WTP settings		$\text {mAP}_{{50}}$ (%)	$\text {mAP}_{{50-90}}$ (%)	F1 (%)	AUC (%)
Levels	1	68.2±1.2	48.0±0.9	63.7±2.5	81.3±3.0
	**2**	**68.8±1.2**	48.2±0.7	**64.1±2.8**	81.6±3.0
	3	**68.8±1.1**	**48.3±0.8**	64.0±2.9	**81.7±2.9**
Learnable	NO	68.4±1.1	47.9±0.8	63.8±3.0	81.2±2.9
	WT	68.1±1.0	47.5±0.4	63.5±2.5	80.8±2.1
	**IWT**	**68.8±1.2**	**48.2±0.7**	**64.1±2.8**	**81.6±3.0**
	ALL	68.5±1.1	48.0±0.7	64.0±3.2	**81.6±3.0**

Bold entries highlight the best-performing metrics

**Fig. 9:**
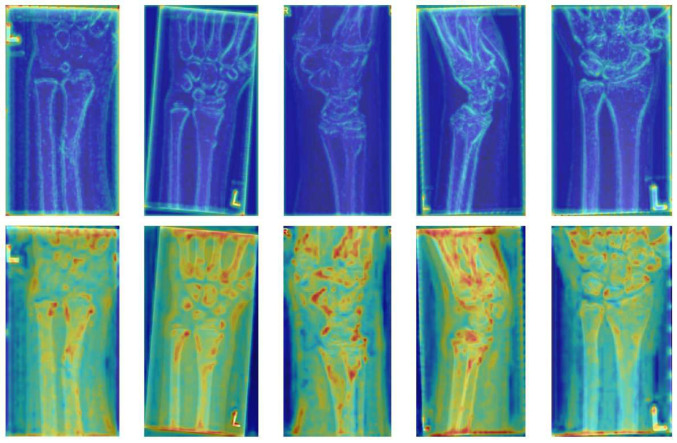
The heatmap visualizations of feature maps before and after WTP. The upper row shows the feature maps prior to WTP, while the lower row exhibits those after WTP. For each sample, we perform a summation operation on all the channels of the feature maps, followed by normalization of the summed results, and then convert them into heatmaps. In these heatmaps, the regions of interest are highlighted in warm colors, while the background is shown in cold colors

#### WTP Under Different Settings

Table 8 illustrates the performance of WTP across various settings. **Levels:** The first three rows demonstrate the performance at different levels. As the level increases, the model performance improves. However, this is accompanied by a corresponding increase in computational complexity. Notably, the performance difference between levels 3 and 2 is marginal. This is because X-ray images are single-channel, which limits the available feature information. Additionally, when low-frequency components undergo successive wavelet transformations, the number of high-frequency components that can be extracted decreases. Consequently, there is no further performance enhancement. **Learnable:** The last four rows present the outcomes when different parameters are learnable, denoted as “NO,” “WT,” “IWT,” and “ALL,” indicating no parameters, only WT parameters, only IWT parameters, and all parameters being learnable, respectively. Our findings indicate that the model performs worst with only WT parameters learnable and best with only IWT parameters learnable. This is attributed to WT involving a downsampling process, while IWT involves upsampling, which is inherently more complex. Furthermore, fixing WT parameters allows the model to effectively learn multi-frequency features.

**Table 9:** Performance of HHM with different duplication times

Duplication times	$\text {mAP}_{{50}}$ (%)	$\text {mAP}_{{50-90}}$ (%)	F1 (%)	AUC (%)	Match time (ms)
[6, 6, 6, 2, 2, 2]	67.9±1.4	48.0±0.7	63.1±2.5	80.6±3.0	5.827±0.108
[7, 6, 5, 4, 3, 2]	68.1±1.2	48.0±0.9	63.2±2.7	81.1±2.7	5.968±0.133
[10, 8, 6, 4, 2, 1]	68.5±1.3	48.3±0.5	63.8±2.9	80.9±3.1	6.042±0.190
[20, 10, 8, 6, 4, 2]	67.9±1.1	47.8±0.9	63.9±2.6	81.0±2.0	6.303±0.295
[15, 12, 10, 8, 6, 3]	67.6±1.2	47.6±0.6	**64.1±2.7**	81.3±2.8	6.301±0.272
[12, 10, 8, 6, 4, 2]	**68.8±1.2**	**48.2±0.7**	**64.1±2.8**	**81.6±3.0**	6.133±0.218

Bold entries highlight the best-performing metrics

To demonstrate the feature extraction ability of WTP, we systematically selected five representative examples. As shown in Fig. 9, we visualized the feature maps as heatmaps. We normalized the heatmap values using the maximum-minimum normalization method, enabling the visualization to reveal the relative intensity of the model’s attention to different features. Our analysis indicates that before WTP implementation, the features predominantly focus on bone edge information, while the bone interior features show significantly lower activation levels. After WTP processing, both the bone interior features and the inter-bone gaps exhibit substantial enhancement. This evidence demonstrates WTP’s effectiveness in filtering out secondary information while amplifying features of primary interest. Notably, the feature map’s upper and lower edge regions exhibit enhanced activation. This phenomenon arises due to the fact that the backbone architecture and WTP module focus on extracting preliminary feature representations, which are subsequently processed by the transformer architecture. By incorporating position encoding, the model adaptively modulates its attention mechanism, effectively reducing focus on edge regions.

#### HHM with Different Duplication Times

In Eq. 13, $w_i$ represents the duplication times for the target at the $i$-th decoder layer, a crucial hyperparameter in HHM. Table 9 displays the results for various duplication times. We primarily tested six configurations, focusing on the differences in duplication times between layers and allocating more or fewer duplications to the shallower layers. Our findings suggest that the model performs optimally with duplication times set to [12, 10, 8, 6, 4, 2]. We hypothesize that this gradual reduction aids the model in progressively eliminating duplicate bounding boxes, leading to a more precise understanding of features and tasks across different layers. We also recommend adjusting duplication times based on the specific object detection task, as the number of bounding boxes varies. Excessive duplications can overwhelm certain layers, while insufficient duplications may result in overfitting.

Since hierarchical matching introduces additional matching costs, to analyze its impact on training time, the last column of Table 9 shows the matching time for a single match under different replication times. The matching time mainly depends on the size of the cost matrix. Given that the number of predicted queries is fixed at 300, the matching time approximately increases linearly with the number of targets. In our task, the number of targets in each image is small (less than 10). Consequently, hierarchical matching does not significantly increase the matching time. Experiments show that when hierarchical matching is not used, the matching time is approximately 5.719 ms. The matching time corresponding to the selected duplication times [12, 10, 8, 6, 4, 2] increases by an average of 0.4 ms. Therefore, the total training time increases by $\frac{0.4\times 17278\times 50\times 0.001}{60}=5.76$ min. This increase is negligible and is acceptable.

**Fig. 10:**
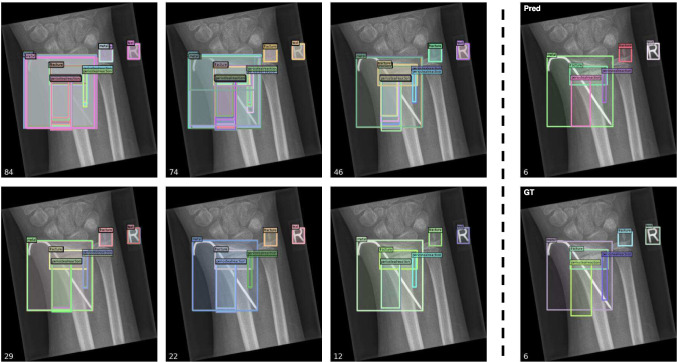
Prediction results from different decoder layers after applying HHM. The left side of the dashed line presents two rows, each containing three predictions. The first row corresponds to the outputs of decoder layers 0 to 2 (with layer 0 being the output of the last encoder layer), and the second row displays the outputs of layers 3 to 5. On the right side of the dashed line, the first row showcases the final predictions, and the second row reveals the ground truth. The bottom-left corner of each image indicates the number of bounding boxes

To further scrutinize the role of HHM, we visualized the predictions of each layer in Fig. 10. The diminishing number of predicted bounding boxes illustrates that HHM functions as expected. The shallower decoder layers produce a greater number of candidate boxes, many of which are duplicates. Subsequent layers systematically eliminate these duplicates and refine the remaining boxes, culminating in the finest candidate boxes. Upon comparison, we observe only slight discrepancies between our predictions and the ground truth. Importantly, our predicted *text* boxes exhibit more precise boundaries, signifying the effectiveness of our model.

## Discussion

Compared with previous YOLO-based object detection algorithms, this study introduces the DETR model for pediatric fracture detection for the first time. This model eliminates the need for post-processing, enabling accurate predictions without the post-processing required by previous YOLO models. This approach has been experimentally proven to be more effective in predicting bounding boxes of various sizes, which is critical for fracture detection. Additionally, this study introduces the WTP module, which better handles high- and low-frequency information in X-ray images compared to CNN modules, thereby preserving the most critical information when mapping the initial features extracted by CNN models to the input of the transformer model. Furthermore, this study proposes the HMM framework, which decouples the prediction targets of different decoder layers during training, resulting in more accurate final predictions. Experiments demonstrate that these improvements are well-suited for pediatric fracture detection tasks. Moreover, we believe that the WH-DETR model is also applicable to similar data, such as X-ray, MRI, and CT scan images, by appropriately adjusting the number of WTP layers and the replication count in HMM, and the proposed model can be adapted to other similar tasks.

Although this study achieves promising results, there are still limitations that can be improved. First, the WTP module tends to identify edge information as important features due to the prominence of edges after image rotation. Although subsequent positional encoding can mitigate this issue, an ideal module should directly ignore such irrelevant information. Second, determining the number of bounding box replications required for each decoder layer in the HMM framework for different tasks requires extensive experimentation, which can be time-consuming. Moreover, the proposed method performs poorly for certain detection categories, such as *boneanomaly* and *softtissue*. These categories feature more complex characteristics, and we believe that incorporating multimodal information, such as patient self-reports, MRI, or CT scans, can further improve detection results for these challenging categories.

## Conclusion

In this study, we presented WH-DETR, a novel end-to-end model designed for detecting pediatric wrist fractures, and introduced two innovative components, WTP and HHM, to bolster its performance. The WTP module enhances detail detection by capturing multi-scale and multi-frequency features, while the HHM framework improves prediction accuracy by disentangling tasks across decoder layers. Experimental results on the GRAZPEDWRI-DX dataset reveal that WH-DETR surpasses existing models in critical metrics. Ablation studies validate the substantial contributions of WTP and HHM to model performance and offervaluable insights into parameter settings for each module. Future endeavors will investigate the applicability of WTP in other medical imaging tasks and refine the HHM framework to accommodate diverse data distributions and detection requirements.

## Data Availability

The dataset (GRAZPEDWRI-DX) used in this study is publicly available at<https://figshare.com/articles/dataset/GRAZPEDWRI-DX/14825193>.

## References

[1] Randsborg PH, Gulbrandsen P, Šaltytė Benth J, Sivertsen EA, Hammer OL, Fuglesang HF, Årøen A: Fractures in Children: Epidemiology and Activity-Specific Fracture Rates. Journal of Bone and Joint Surgery, 95:e42, 10.2106/jbjs.l.00369, 2013 10.2106/JBJS.L.00369 23553305

[2] Bamford R, Walker DM: A qualitative investigation into the rehabilitation experience of patients following wrist fracture. Hand Therapy, 15:54-61, 10.1258/ht.2010.010013, 2010

[3] Kraus R, Wessel L: The Treatment of Upper Limb Fractures in Children and Adolescents. Deutsches Ärzteblatt international, 107:903-910, 10.3238/arztebl.2010.0903, 2010 10.3238/arztebl.2010.0903 PMC3023154 21249137

[4] Rimmer A: Radiologist shortage leaves patient care at risk, warns royal college. BMJ, 359, https://www.bmj.com/content/359/bmj.j4683, 2017 10.1136/bmj.j4683 29021184

[5] Freed HA, Shields NN: Most frequently overlooked radiographically apparent fractures in a teaching hospital emergency department. Annals of Emergency Medicine, 13:900-904, 10.1016/s0196-0644(84)80666-6, 1984 10.1016/s0196-0644(84)80666-6 6476514

[6] Hedström EM, Svensson O, Bergström U, Michno P: Epidemiology of fractures in children and adolescents: Increased incidence over the past decade: a population-based study from northern Sweden. Acta Orthopaedica, 81:148-153, 10.3109/17453671003628780, 2010 10.3109/17453671003628780 PMC2856220 20175744

[7] Burki TK: Shortfall of consultant clinical radiologists in the UK. The Lancet Oncology, 19:e518, 10.1016/s1470-2045(18)30689-2, 2018 10.1016/S1470-2045(18)30689-2 30220623

[8] Smalley C: Most Frequently Missed Fractures in the Emergency Department. The Journal of Emergency Medicine, 41:110, 10.1016/j.jemermed.2011.05.005, 2011

[9] Anthony Brinton Wolbarst: Looking Within: How X-Ray, CT, MRI, Ultrasound, and Other Medical Images Are Created, and How They Help Physicians Save LivesBerkeley:University of California Press, 2000

[10] Nasser M, Puig D, Moreno A: Improvement of Mass Detection In Breast X-Ray Images Using Texture Analysis Methods. Frontiers in Artificial Intelligence and Applications, 269, 10.3233/978-1-61499-452-7-159, 2014

[11] Choi JW, Cho YJ, Lee S, Lee J, Lee S, Choi YH, Cheon JE, Ha JY: Using a Dual-Input Convolutional Neural Network for Automated Detection of Pediatric Supracondylar Fracture on Conventional Radiography. Investigative Radiology, 55:101-110, 10.1097/rli.0000000000000615, 2019 10.1097/RLI.0000000000000615 31725064

[12] Tanzi L, Vezzetti E, Moreno R, Aprato A, Audisio A, Massè A: Hierarchical fracture classification of proximal femur X-Ray images using a multistage Deep Learning approach. European Journal of Radiology, 133:109373, 10.1016/j.ejrad.2020.109373, 2020 10.1016/j.ejrad.2020.109373 33126175

[13] Chung SW, Han SS, Lee JW, Oh KS, Kim NR, Yoon JP, Kim JY, Moon SH, Kwon J, Lee HJ, Noh YM, Kim Y: Automated detection and classification of the proximal humerus fracture by using deep learning algorithm. Acta Orthopaedica, 89:468-473, 10.1080/17453674.2018.1453714, 2018 10.1080/17453674.2018.1453714 PMC6066766 29577791

[14] Ju RY, Cai W: Fracture detection in pediatric wrist trauma X-ray images using YOLOv8 algorithm. Scientific Reports, 13, 10.1038/s41598-023-47460-7, 2023 10.1038/s41598-023-47460-7 PMC10654405 37973984

[15] Chun-Tse Chien, Rui-Yang Ju, Kuang-Yi Chou, Jen-Shiun Chiang: YOLOv9 for fracture detection in pediatric wrist trauma X-ray images. Electronics Letters, 60:e13248, https://digital-library.theiet.org/doi/abs/10.1049/ell2.13248, 2024

[16] Dibo R, Galichin A, Astashev P, Dylov DV, Rogov OY: DeepLOC: Deep Learning-Based Bone Pathology Localization and Classification in Wrist X-Ray Images. Analysis of Images, Social Networks and Texts, 199-211, 10.1007/978-3-031-54534-4_14, 2024

[17] Blüthgen C, Becker AS, Vittoria de Martini I, Meier A, Martini K, Frauenfelder T: Detection and localization of distal radius fractures: Deep learning system versus radiologists. European Journal of Radiology, 126:108925, 10.1016/j.ejrad.2020.108925, 2020 10.1016/j.ejrad.2020.108925 32193036

[18] Ahmed A, Imran AS, Manaf A, Kastrati Z, Daudpota SM: Enhancing wrist abnormality detection with YOLO: Analysis of state-of-the-art single-stage detection models. Biomedical Signal Processing and Control, 93:106144, 10.1016/j.bspc.2024.106144, 2024

[19] Zech JR, Carotenuto G, Igbinoba Z, Tran CV, Insley E, Baccarella A, Wong TT: Detecting pediatric wrist fractures using deep-learning-based object detection. Pediatric Radiology, 53:1125-1134, 10.1007/s00247-023-05588-8, 2023 10.1007/s00247-023-05588-8 36650360

[20] Redmon J, Divvala S, Girshick R, Farhadi A: You Only Look Once: Unified, Real-Time Object Detection. 2016 IEEE Conference on Computer Vision and Pattern Recognition (CVPR), 779-788, 10.1109/cvpr.2016.91, 2016

[21] Lin TY, Maire M, Belongie S, Hays J, Perona P, Ramanan D, Dollár P, Zitnick CL: Microsoft COCO: Common Objects in Context. Computer Vision - ECCV 2014, 740-755, 10.1007/978-3-319-10602-1_48, 2014

[22] Reddy Dirisala S, Padidem D: Comparative Study of the Different Object Detection Algorithms: YOLOv4, SSD, and RCNN based on Accuracy and Speed. International Journal of Science and Research (IJSR), 12:1560-1565, 10.21275/sr231020230143, 2023

[23] Wang CY, Bochkovskiy A, Liao HYM: YOLOv7: Trainable Bag-of-Freebies Sets New State-of-the-Art for Real-Time Object Detectors. 2023 IEEE/CVF Conference on Computer Vision and Pattern Recognition (CVPR), 7464-7475, 10.1109/cvpr52729.2023.00721, 2023

[24] Sohan, Mupparaju,Sai Ram, Thotakura, Rami Reddy, Ch. Venkata: A Review on YOLOv8 and Its Advancements. Data Intelligence and Cognitive Informatics, 529-545, 10.1007/978-981-99-7962-2_39, 2024

[25] Wang CY, Yeh IH, Mark Liao HY: YOLOv9: Learning What You Want to Learn Using Programmable Gradient Information. Computer Vision - ECCV 2024, 1-21, 10.1007/978-3-031-72751-1_1, 2024

[26] Vaswani A, Shazeer N, Parmar N, Uszkoreit J, Jones L, Gomez AN, Kaiser Polosukhin I: Attention is all you need. Proceedings of the 31st International Conference on Neural Information Processing Systems, 6000-6010, https://dl.acm.org/doi/10.5555/3295222.3295349, 2017

[27] Xi X, Huang Z, Wu Y, Li J: End-to-End Object Detection with YOLOF. 10.2139/ssrn.4481769, 2023

[28] Liang T, Zeng G: FCM-DETR: An Efficient End-to-End Fire Smoke and Human Detection based on Deformable DETR. 10.20944/preprints202405.1823.v1, 2024 10.3390/s24134077 PMC11244274 39000856

[29] Geng H, Jiang J, Shen J, Hou M: Cascading Alignment for Unsupervised Domain-Adaptive DETR with Improved DeNoising Anchor Boxes. Sensors, 22:9629, 10.3390/s22249629, 2022 10.3390/s22249629 PMC9783326 36560000

[30] Lin Y, Yuan Y, Zhang Z, Li C, Zheng N, Hu H: DETR Does Not Need Multi-Scale or Locality Design. 2023 IEEE/CVF International Conference on Computer Vision (ICCV), 6522-6531, 10.1109/iccv51070.2023.00602, 2023

[31] Li C, Zhang J, Huo B, Xue Y: DHQ-DETR: Distributed and High-Quality Object Query for Enhanced Dense Detection in Remote Sensing. Remote Sensing, 17:514, 10.3390/rs17030514, 2025

[32] Nagy E, Janisch M, Hržić F, Sorantin E, Tschauner S: A pediatric wrist trauma X-ray dataset (GRAZPEDWRI-DX) for machine learning. Scientific Data, 9, 10.1038/s41597-022-01328-z, 2022 10.1038/s41597-022-01328-z PMC9122976 35595759

[33] Guan B, Yao J, Zhang G, Wang X: Thigh fracture detection using deep learning method based on new dilated convolutional feature pyramid network. Pattern Recognition Letters, 125:521-526, 10.1016/j.patrec.2019.06.015, 2019

[34] Wang M, Yao J, Zhang G, Guan B, Wang X, Zhang Y: ParallelNet: multiple backbone network for detection tasks on thigh bone fracture. Multimedia Systems, 27:1091-1100, 10.1007/s00530-021-00783-9, 2021

[35] Virasova A, Klimov D, Khromov O, Gubaidullin I, Oreshko V: Rich feature hierarchies for accurate object detection and semantic segmentation. Radioengineering, 115-126, 10.18127/j00338486-202109-11, 2021

[36] Liu J, Wang D, Lu L, Wei Z, Kim L, Turkbey EB, Sahiner B, Petrick NA, Summers RM: Detection and diagnosis of colitis on computed tomography using deep convolutional neural networks. Medical Physics, 44:4630-4642, 10.1002/mp.12399, 2017 10.1002/mp.12399 PMC5603218 28594460

[37] Cai Z, Vasconcelos N: Cascade R-CNN: Delving Into High Quality Object Detection. 2018 IEEE/CVF Conference on Computer Vision and Pattern Recognition, 6154-6162, 10.1109/cvpr.2018.00644, 2018

[38] Wu HZ, Yan LF, Liu XQ, Yu YZ, Geng ZJ, Wu WJ, Han CQ, Guo YQ, Gao BL: The Feature Ambiguity Mitigate Operator model helps improve bone fracture detection on X-ray radiograph. Scientific Reports, 11, 10.1038/s41598-021-81236-1, 2021 10.1038/s41598-021-81236-1 PMC7810849 33452403

[39] Xie S, Girshick R, Dollar P, Tu Z, He K: Aggregated Residual Transformations for Deep Neural Networks. 2017 IEEE Conference on Computer Vision and Pattern Recognition (CVPR), 10.1109/cvpr.2017.634, 2017

[40] Wang C, Zhong C: Adaptive Feature Pyramid Networks for Object Detection. IEEE Access, 9:107024-107032, 10.1109/access.2021.3100369, 2021

[41] Liu Z, Lin Y, Cao Y, Hu H, Wei Y, Zhang Z, Lin S, Guo B: Swin Transformer: Hierarchical Vision Transformer using Shifted Windows. 2021 IEEE/CVF International Conference on Computer Vision (ICCV), 9992-10002, 10.1109/iccv48922.2021.00986, 2021

[42] Ju RY, Chien CT, Lin CM, Chiang JS: Global Context Modeling in YOLOv8 for Pediatric Wrist Fracture Detection. 2024 International Symposium on Intelligent Signal Processing and Communication Systems (ISPACS), 1-5, 10.1109/ispacs62486.2024.10869064, 2024

[43] Chien CT, Ju RY, Chou KY, Xieerke E, Chiang JS: YOLOv8-AM: YOLOv8 Based on Effective Attention Mechanisms for Pediatric Wrist Fracture Detection. IEEE Access, 1-1, 10.1109/access.2025.3549839, 2025

[44] Wang Y, Zhang X, Yang T, Sun J: Anchor DETR: Query Design for Transformer-Based Detector. Proceedings of the AAAI Conference on Artificial Intelligence, 36:2567-2575, 10.1609/aaai.v36i3.20158, 2022

[45] Meng D, Chen X, Fan Z, Zeng G, Li H, Yuan Y, Sun L, Wang J: Conditional DETR for Fast Training Convergence. 2021 IEEE/CVF International Conference on Computer Vision (ICCV), 3631-3640, 10.1109/iccv48922.2021.00363, 2021

[46] Dai X, Chen Y, Yang J, Zhang P, Yuan L, Zhang L: Dynamic DETR: End-to-End Object Detection with Dynamic Attention. 2021 IEEE/CVF International Conference on Computer Vision (ICCV), 2968-2977, 10.1109/iccv48922.2021.00298, 2021

[47] Li F, Zhang H, Liu S, Guo J, Ni LM, Zhang L: DN-DETR: Accelerate DETR Training by Introducing Query DeNoising. 2022 IEEE/CVF Conference on Computer Vision and Pattern Recognition (CVPR), 10.1109/cvpr52688.2022.01325, 2022 10.1109/TPAMI.2023.3335410 38019624

[48] Zhao Y, Lv W, Xu S, Wei J, Wang G, Dang Q, Liu Y, Chen J: DETRs Beat YOLOs on Real-time Object Detection. 2024 IEEE/CVF Conference on Computer Vision and Pattern Recognition (CVPR), 16965-16974, 10.1109/cvpr52733.2024.01605, 2024

[49] Zhao C, Sun Y, Wang W, Chen Q, Ding E, Yang Y, Wang J: MS-DETR: Efficient DETR Training with Mixed Supervision. 2024 IEEE/CVF Conference on Computer Vision and Pattern Recognition (CVPR), 17027-17036, 10.1109/cvpr52733.2024.01611, 2024

[50] Daubechies I: Society for Industrial and Applied Mathematics, https://epubs.siam.org/doi/abs/10.1137/1.9781611970104, 1992

[51] Wang T, Lu C, Sun Y, Yang M, Liu C, Ou C: Automatic ECG Classification Using Continuous Wavelet Transform and Convolutional Neural Network. Entropy, 23:119, 10.3390/e23010119, 2021 10.3390/e23010119 PMC7831114 33477566

[52] Alhussainy AMH: A New Pooling Layer based on Wavelet Transform for Convolutional Neural Network. Journal of Advanced Research in Dynamical and Control Systems, 24:76-85, 10.5373/jardcs/v12i4/20201420, 2020

[53] Huang H, He R, Sun Z, Tan T: Wavelet-SRNet: A Wavelet-Based CNN for Multi-scale Face Super Resolution. 2017 IEEE International Conference on Computer Vision (ICCV), 1698-1706, 10.1109/iccv.2017.187, 2017

[54] Saragadam V, LeJeune D, Tan J, Balakrishnan G, Veeraraghavan A, Baraniuk RG: WIRE: Wavelet Implicit Neural Representations. 2023 IEEE/CVF Conference on Computer Vision and Pattern Recognition (CVPR), 18507-18516, 10.1109/cvpr52729.2023.01775, 2023

[55] Finder SE, Amoyal R, Treister E, Freifeld O: Wavelet Convolutions for Large Receptive Fields. Computer Vision - ECCV 2024, 10.1007/978-3-031-72949-2_21, 2025

[56] He K, Zhang X, Ren S, Sun J: Deep Residual Learning for Image Recognition. 2016 IEEE Conference on Computer Vision and Pattern Recognition (CVPR), 10.1109/cvpr.2016.90, 2016

[57] Gal R, Hochberg DC, Bermano A, Cohen-Or D: SWAGAN: a style-based wavelet-driven generative model. ACM Transactions on Graphics, 40:1-11, 10.1145/3476576.3476707, 2021

[58] Jia D, Yuan Y, He H, Wu X, Yu H, Lin W, Sun L, Zhang C, Hu H: DETRs with Hybrid Matching. 2023 IEEE/CVF Conference on Computer Vision and Pattern Recognition (CVPR), 19702-19712, 10.1109/cvpr52729.2023.01887, 2023

[59] Loshchilov I, Hutter F: Decoupled weight decay regularization. arXiv preprint arXiv:1711.05101, 10.48550/arXiv.1711.05101, 2017

